# Proteomic Serum Biomarkers and Their Potential Application in Cancer Screening Programs

**DOI:** 10.3390/ijms11114175

**Published:** 2010-10-26

**Authors:** Anouck Huijbers, Berit Velstra, Tim J. A. Dekker, Wilma E. Mesker, Yuri E. M. van der Burgt, Bart J. Mertens, André M. Deelder, Rob A. E. M. Tollenaar

**Affiliations:** 1 Department of Surgery, Leiden University Medical Center (LUMC), Albinusdreef 2, 2333 ZA Leiden, The Netherlands; E-Mails: a.huijbers@lumc.nl (A.H.); b.velstra@lumc.nl (B.V.); w.e.mesker@lumc.nl (W.E.M.); 2 Department of Parasitology, Biomolecular Mass Spectrometry Unit, Leiden University Medical Center (LUMC), Albinusdreef 2, 2333 ZA Leiden, The Netherlands; 3 Department of Medical Statistics, Leiden University Medical Center (LUMC), Albinusdreef 2, 2333 ZA Leiden, The Netherlands

**Keywords:** mass spectrometry, biomarker, MALDI-TOF, SELDI-TOF, colorectal cancer, breast cancer, screening, proteomics, magnetic beads

## Abstract

Early diagnosis of cancer is of pivotal importance to reduce disease-related mortality. There is great need for non-invasive screening methods, yet current screening protocols have limited sensitivity and specificity. The use of serum biomarkers to discriminate cancer patients from healthy persons might be a tool to improve screening programs. Mass spectrometry based proteomics is widely applied as a technology for mapping and identifying peptides and proteins in body fluids. One commonly used approach in proteomics is peptide and protein profiling. Here, we present an overview of profiling methods that have the potential for implementation in a clinical setting and in national screening programs.

## 1. Introduction

Population wide screening programs are used to detect early stage cancer to enable early intervention and reduce morbidity and mortality. Ideally screening tests have to be highly specific, sensitive, cost-effective and non-invasive. The development of new screening methods has become important due to an increasing incidence, as is the case for colorectal cancer (CRC). In addition, novel screening strategies aim at improved sensitivity and specificity in case of breast cancer. Advanced cancer has a poor survival, whereas when diagnosed at an early stage, survival is relatively good [1]. Early detection will identify cancer when it is still localized and curable, preventing not only mortality, but also reducing morbidity and costs [1–5]. The use of serum biomarkers as an indicator of disease in cancer screening programs could provide a promising alternative to existing methods.

A biomarker, or biological marker, is a biomolecule that can be used as an indicator of a disease, based on abnormal presence, absence or changes in genes, RNA, proteins or metabolites. In this manuscript we will discuss protein biomarkers. The ideal biomarker is both highly specific and sensitive. For screening programs, the required measurements have to be reliable, robust, fast, and economical. The material containing the marker(s) should be easily obtainable and have a patient-friendly application. In this respect, body fluids such as serum are suitable sources of biomarkers. Possible applications are (early) detection, prediction of survival and prediction and monitoring of response to therapy. Here we focus on the use of protein biomarkers for early cancer detection.

The translation of the DNA code results in protein expression. In contrast to the genome, the proteome reflects a more dynamic state of the cell [6]. During transformation of a normal cell into a neoplastic cell, distinct changes occur at the protein level, including altered expression, different protein posttranslational modifications, changes in specific activity and inappropriate localization, all of which may affect cellular function [4,7]. By comparing the protein patterns, *i.e.*, profiles, in serum from patients with cancer with those obtained from healthy individuals, proteins that are the most discriminating can be classified. The resulting protein fingerprint has the potential to identify a person with cancer. Mass spectrometry (MS) has proven to be a powerful tool in obtaining such protein fingerprints due to its high sensitivity and specificity. In fact, proteomic research has benefitted enormously from developments in MS technology and has evolved into a new field that is referred to as MS-based proteomics [8]. Whereas proteomics aims for the full identification and quantification of all expressed proteins, profiling strategies usually are applied on sub-sets of the proteome. Importantly, all steps in MS based profiling methods can be fully automated allowing high sample throughput and standardization [9]. In finding biomarkers for early cancer detection, the content of this review is limited to results obtained from protein profiling efforts.

## 2. Screening for Breast Cancer and Colorectal Cancer

### 2.1. Breast Cancer

Breast cancer is the most commonly diagnosed malignancy in women with over one million new cases in the world each year [10]. With an increasing lifetime risk, currently estimated at one in eight, it is a leading cause of cancer-related morbidity and mortality. Despite increasing incidence rates, annual mortality rates from breast cancer have decreased over the last decade [11]. Reasons for this decline include precise diagnosis, increased number of women receiving tailor made treatment, such as extensive use of tamoxifen, and the use of chemotherapy and early detection through widespread mammography screening [10,12].

Mammography is currently the most important tool in screening and early detection of breast cancer [10]. In many countries, mammography is used as a population based screening method in women over 50 years of age. However, up to 20% of new breast cancer incidents are not detected by this method [13–15]. Furthermore, only one out of three lesions positively detected using mammography turns out to be malignant. Mammography is also used as a screening tool in young women with a high familiar risk or with a genetic predisposition. In this group the detection rate is only 40%, mainly because of the dense breast tissue [16,17]. Adding MRI to mammography screening for these at risk patients has good potential to detect mammographically occult cancers but this expensive imaging technique does not reliably distinguish benign from malignant findings and has a high false positive rate [18–20]. Consequently, MRI and also mammography screening can lead to overdiagnosis and overtreatment [18,21], indicating a need for novel molecular markers that might improve specificity and sensitivity for early detection of breast cancers, suitable for population screening or more intensified screening programs for young women with an increased risk.

### 2.2. Colorectal Cancer

Colorectal cancer (CRC) is among the most common malignancies and remains a leading cause of cancer-related morbidity and mortality. There are approximately one million new cases of CRC per year worldwide [22]. Although the incidence of CRC is fortunately decreasing in the United States [9,23], in most other countries the incidence rates are increasing, particularly due to the increase in total population and aging of the current population. In Asia, Eastern Europe, Israel, and Puerto Rico the increase is most dominant. Colorectal cancer arises from a multistep sequence of genetic alterations that results in the transformation of normal mucosa to a precursor adenoma and ultimately to carcinoma. Early detection appears to be the most influential factor to reduce disease related mortality and treatment related morbidity [23,24]. Unfortunately, at this moment only about 37% of CRC remain localized at the time of diagnosis [25]. Survival in CRC is directly related to the stage of the disease at the time of diagnosis. When cancer is found early at localized stage (stage I), five-year survival is approximately 95% [9,26]; whereas the overall five-year survival rate of CRC with distance metastasis to distance is less than five percent. Early detection by population wide screening programs thus becomes more important.

Access to screening programs varies throughout the world, from population programmatic screening in developed countries to regional level screening programs or the opportunity of having a screening test when entering a health care system. Screening programs in most countries include average risk individuals aged between 50 and 75 years [22] and vary widely in screening incidence as well as in the method of choice.

Currently available tests used for screening include guaiac-based fecal occult blood test (gFOBT), immunochemical fecal occult blood test (iFOBT), colonoscopy and flexible sigmoidoscopy (FS). Other less commonly used tests are stool DNA testing (sDNA), computed tomography colonography (CTC) and double-contrast barium enema (DCBE).

Due to its low costs and easy access, the most frequently used screening method is gFOBT. It detects the peroxidase reaction of hemoglobin. Disadvantages are the false–positive rates which make dietetic provisions necessary and low sensitivity rates from 20–40% [27].

With iFOBT, no dietetic restrictions are necessary because it only reacts to human hemoglobin. A wide range of qualitative and quantitative tests are presently available, with varying levels of sensitivity and specificity. With only one test, sensitivity rates are approximately 65%; when repeated every two years, sensitivity increases to 80–90% [28,29].

FS is an endoscopic examination with maximum reach to the splenic flexure. Its sensitivity is about 60–70% for adenomas and CRC [30,31]. Unlike FS, colonoscopy also detects lesions in the proximal colon. Its biggest advantage is the possibility of removing pathological lesions within a single examination. The sensitivity in detecting both adenomas and carcinomas seems to be high but data from prospective, randomized trials are limited. Also it is an invasive method with a higher risk of serious adverse events than for FS, respectively 3–5% compared to 0% to 0.03% [30,32,33]. To implement colonoscopy into national screening programs, a huge increase in care capacities would be necessary.

sDNA examines the stool for the presence of abnormal DNA. The test sensitivity for CRC ranges from 52% to 91% [27,34–36]. Another disadvantage is its high price.

CTC shows lesions in the colorectum by reconstructing two- and three-dimensional images. To date, no studies have been published assessing reduction in CRC incidence or mortality. DCBE shows the entire colorectum, although with significantly lower sensitivity and specificity than colonoscopy or CTC. The percentage of undetected carcinomas is up to 22% [33].

No available CRC screening test is yet perfect, either for cancer detection or adenoma detection. Each test has associated limitations and risks. There is a great need for alternative, non-invasive methods with high sensitivity and specificity rates, easily available and cost effective. Use of MS based proteomic serum biomarkers could form a specific, more sensitive and less invasive alternative.

## 3. Workflow in Proteomic Profiling

### 3.1. Blood Sample Preparation

Human blood is a suitable source of proteins and can be obtained in a relatively easy fashion. Both plasma and serum samples, obtained from whole blood, have been used in biomarker discovery studies. Serum resembles plasma in composition but lacks the coagulation factors. Although serum is preferred for many tests because the anticoagulants in plasma can sometimes interfere with the method, plasma seems to be more stable than serum and more suitable for analysis of the low-molecular-weight proteome. It has been reported by various authors that protein profiles obtained from plasma and serum differ and unfortunately at this time it would appear that insufficient information is available to decide whether serum or plasma should be preferentialized in MS-based proteomics studies aiming for biomarker discovery. While most studies have been carried out using serum, further research on this topic is required. A temporary solution would be to use both, however this would complicate data analysis and require longer processing times.

### 3.2. Standardization

As is the case for all diagnostic tools in a clinical setting, MS based proteomic profiles should be precise and accurate, and the methodology needs to be robust and reproducible. Some critics have argued that discriminating peaks are influenced by various factors. Possible confounding factors can be categorized into three sources of variation and bias: biological variation, pre-analytical variation and analytical reproducibility. Examples of biological variation are race, age, diet, smoking, but also stress, drugs and general physical conditions [37–39]. To date, no studies have been reported taking into account these latter aspects. Some groups have reported data on the effects of different sample preparation procedures. In all studies the importance of sample handling was indicated; *i.e.*, the time between blood sampling and serum centrifugation. A delay of two to four hours seems to be acceptable. De Noo *et al*. analyzed pre-analytical variables and reproducibility on a matrix-assisted laser desorption ionization time-of-flight (MALDI-TOF) approach.

It is now generally recognized that standardized sample collection is required for clinical studies [40,41]. In addition, it is recommended that the number of freeze/thaw cycles is kept as low as possible. Finally, it was found that circadian rhythm was not an influencing factor, in other words samples can be collected at any time of the day. Both the acceptable delay time before serum centrifugation and the ability to collect samples throughout the day increases future clinical applicability [38].

The Human Proteome Organisation (HUPO) is an international scientific organization representing and promoting proteomics through international cooperation and collaboration by fostering the development of new technologies, techniques and training. (www.hupo.org). For this review, interesting HUPO initiatives are the HUPO Plasma Proteome Project (HPPP) and Human Proteome Project (HPP). A goal of the first initiative is to organize more standardized procedures regarding the collection and measuring of the samples and data processing. An overview of the HPPP results from the pilot phase with 35 collaborating laboratories and multiple analytical groups, generating a core dataset of 3020 proteins and a public database [42]. The mission of the HPP is to characterize all 21,000 genes of the known genome, thus generating a map of the protein based molecular architecture of the human body and providing a resource to help elucidate biological and molecular function and advance diagnosis and treatment of diseases[43].

### 3.3. Clean-up Procedure

Human body fluids such as serum are complex mixtures of salts, lipids, peptides and proteins. To carry out a repeatable and robust mass spectrometric analysis of proteins in body fluids, a clean-up or extraction procedure is required [44]. In general, protein separation techniques are based on different physical properties of a protein, such as size, iso-electric point, solubility and affinity. Furthermore, the use of a specific agent to capture proteins enriches the sample and thus improves the lower limits of detection. Obviously, enrichment procedures are of great value to capture so-called low-abundance proteins. Unfortunately, low-abundance proteins are often not circulating freely but are specifically bound to high-abundance proteins, such as albumin. As a result proteins present at low concentration can be lost in depletion methods [9].

### 3.4. Functionalized Magnetic Beads

In the last decade, multiple studies have been carried out using magnetic beads as a method for offline serum peptide/protein capture [38,45–47]. Magnetic beads are uniform beads specifically designed for quick manual or automated fractionation of proteins or peptides from complex biological samples. This solid-phase extraction (SPE) procedure is quick and simple, sample preparation occurs without the need for laborious pipetting and centrifugation. As mentioned earlier, protein separation techniques are based on different physical properties of a protein. Materials known from these different chromatographic platforms are coupled to the surface of spherical magnetic beads. Magnetic beads most applied in studies are WCX (weak cation exchange), RPC18 beads (reversed phase) and C8. WCX beads separate proteins based on charge, whereas RPC18 beads separate proteins and peptides via strong hydrophobic interaction[48].

### 3.5. Automation

The manual fractionation and processing steps are tedious and time consuming to perform. Automation ensures reproducibility and facilitates high-throughput performance necessary for large scale studies. In the last few years our study group developed a reliable automated technique that is specially designed for high-throughput sample handling, *i.e.*, processing hundreds of serum samples per day. The activation, wash and desorption steps of WCX and RPC18 beads are based on the manufacturers protocol, with adjustments to allow for optimal implementation on a 96-channel Hamilton STARplus^®^ pipetting robot. With this liquid handling robot, the whole serum peptide/protein capture procedure is automated. Spotting onto a MALDI target plate is carried out in quadruplicate using the same robot.

### 3.6. MALDI-TOF Mass Spectrometry

Mass spectrometry (MS) is the method of choice for the analysis of proteins in serum [8]. A mass spectrometer separates peptides or proteins according to their mass-to-charge ratio. A mass spectrometer consists of an ion source, a mass analyzer and a detector. There are several types of mass spectrometers, the one mostly used in profiling strategies is MALDI-TOF. To carry out a MALDI-TOF mass analysis, a small amount of specimen containing peptides and proteins is dried on a target plate after mixing with a light-absorbing matrix. MALDI-TOF MS is a rapid biomarker discovery tool that allows high-throughput screening through automated sample processing and profiling.

### 3.7. SELDI-TOF

As an alternative, surface enhanced laser desorption ionization-time-of-flight (SELDI-TOF) can be used [49]. In SELDI-TOF a surface modified with a chemical functionality on a chip is used. A sample clean-up is then carried out on this chip similar to workup with functionalized magnetic beads. Some proteins in the sample bind to the chip surface, while the others are removed by washing. After washing the spotted sample, the matrix is applied to the surface and allowed to crystallize with the sample peptides. Common surfaces include CM10 (weak-positive ion exchange), H50 (hydrophobic surface, similar to C6C12 reverse phase chromatography), IMAC30 (metal-binding surface), and Q10 (strong anion exchanger). Surfaces can also be functionalized with antibodies, other proteins or DNA. Samples spotted on a SELDI surface are mass analyzed using TOF-MS as used in MALDI-TOF.

Combined with magnetic bead fractionation, MALDI-TOF has higher throughput than SELDI-TOF and is more sensitive, as spherical particles have larger surface areas and higher binding capacity than chips. Thus, in SELDI-TOF more serum is necessary for analysis.

### 3.8. Data Analysis

Next to standardized sample collection and preparation protocols, the data analysis is of major importance. In 2008, our group organized a competition on clinical mass spectrometry based proteomic diagnosis. Eleven international statistical groups participated and constructed a diagnostic classification rule for allocation of future patients on a blinded calibration set. This classification rule was then tested on a blinded validation set. A variety of statistical methods was used to create a classification rule. Mertens and co-workers described a method in which classification error rates were estimated and validated based on a classical Fisher linear discriminant analysis through complete double cross validation [50]. Each sample was assigned to the group for which the probability was highest. Other groups, for example, used the random forest classification method, or a three-step approach with ranking of the mass/charge values using random forests, then grouping into new variables and finding a discriminating rule by penalized logistic regression. For further details and additional statistical methods see http://www.bepress.com/sagmb/vol7/iss2. This competition showed that a discriminating profile could be created independently of the chosen statistics with consistent results of 80% accuracy [51].

## 4. Potential Mass Spectrometry Derived Biomarkers

### 4.1. Early Detection of Breast Cancer

Several studies have used mass spectrometry (MS) on serum samples in an attempt to find biomarkers for early diagnosis of breast cancer using the SELDI-TOF [52–58] or MALDI-TOF approach [45]. All studies were case controlled, except for the study by Mathelin *et al*. The various studies included sample sizes from 40 to 109 cancer patients with control groups of equal size. Results were encouraging with high sensitivity and specificity rates, varying from 80 to 100%. Several discriminatory peaks were described, such as a peak at 8.9 kDA [52,53,56,59], 4.3 kDa [56–58] and one at 8.1 kDa [53,56].

However, the reproducibility of these results has been questioned. Li *et al*. identified three peaks associated with breast cancer, termed BC1 (4.3 kDa), BC2 (8.1 kDa) and BC3 (8.9 kDA) [56]. The combination of these three biomarkers allowed differentiation of cancer patients and non-cancer controls with a sensitivity of 93% and specificity of 91%. Mathelin *et al*. tried to validate these three biomarkers in a set of 49 breast cancer patients and 13 patients with benign breast tumors and 27 controls [58]. Although, both of these studies used SELDI-TOF and nickel-loaded proteinchip arrays, Mathelin *et al*. could not identify the BC2 peak in their patient series. A combination of BC1 and BC3 could only identify 45% of all breast cancer patients successfully. This is a somewhat disappointing result that might indicate that results obtained in one laboratory are difficult to reproduce in another laboratory or setting. Although limited information concerning handling protocols was provided in the reports of these two studies, differences in methods might have been responsible for this lack of reproducibility [60]. Remarkably, another study found that the peak at 8.9 kDa was decreased, whereas in other studies this peak was increased [61]. Even a follow-up study by the same group could not reproduce the BC1 peak [62].

All described differences can be due to modification of peptides or proteins from the moment of sample collection to freezing, which has been described [63]. Some of these studies used different methods with regard to time between collection and freezing, time of centrifugation, and storage freezing temperature, which may well lead to variability in outcome. Results by Fan *et al*. were more optimistic. This study tested a classification model after initial identification in a different patient group. On a blinded patient population, this model had high sensitivity and specificity (96.45% and 94.87% respectively) [53], indicating a good reproducibility if MS is performed under the exact same conditions (Table 1).

Like in colorectal cancer, the size of the investigated groups was relatively small. Some studies found MS differentiated benign from malignant abnormalities [52], but most studies used healthy people as controls which obviously is not representative of the general patient population.

### 4.2. Early Detection of CRC

Mass spectrometry has been applied for the development of tests for early diagnosis of CRC in several studies [64–70]. All of these were case-control studies and, so far, no prospective or randomized studies have been reported. Published studies reported promising results and underline the potential of mass spectrometry for early diagnostics. Patients diagnosed at several stages of colorectal cancer were included these studies (Dukes stage I to IV). Although for all mentioned studies serum samples were used, the applied methods differed. Only de Noo *et al.* used MALDI-TOF MS in combination with C8 magnetic beads, while all others used the SELDI-TOF system with varying detection chips. For instance, Engwegen *et al.* found the best results by using CM10 chips, while Liu *et al*. compared obtained serum profiles with several chips and found the best results using the IMAC30 chip with the SELDI-TOF system. More research has to be done to optimize pre-analytical and detection variables. However, Ward *et al.* and Liu *et al.* found reproducible results when identical methods and materials were used. Various studies show many variations in methods for storage and handling of the serum samples, the time period between sample collection and freezing, and samples being stored at different temperatures.

The aforementioned studies used discriminant analysis to discriminate between cancer patients and healthy controls. Interestingly, several peaks were repeatedly found in multiple studies signifying their potential as a biomarker. For instance, a peak at m/z ratio 8940 Da (identified as complement protein C3a-desArg) was found by Ward *et al.*, Habermann *et al.*, Zhao *et al.* and Yu *et al*. Another peak at 5911 Da was used as a discriminating peak both by Yu *et al.* and by Chen *et al*. Most studies tried several combinations of significant peaks and used those to identify cancer patients. All studies were capable of achieving sensitivity and specificity values of around 90% or higher. However, we have to be cautious since these results might be overoptimistic. Since some of the algorithms were tested on the same group of patients which was used to create the algorithm, results might be biased. Also, relatively small groups were used in these studies (40 to 60 colorectal cases with control groups consisting of a similar size of healthy controls). Validation of these results on a larger and independent patient group is therefore necessary. Some of the published studies used a (small) independent group for validation of the sensitivity and specificity [65,68,71]. Engwegen *et al*. validated their classification tree on independent patient samples, from which a test sensitivity and specificity of 66.7% and 89.5% were found. Liu *et al*. found a sensitivity of 95% and specificity of 94.87% when testing their biomarkers on a set of 60 cancer patients and 39 healthy subjects (Table 2).

However, the reason these techniques are being developed is for screening in patient populations where the *a priori* chance of having colon cancer is much smaller than in the patient series in these studies. With a lower *a priori* chance, the positive predictive value will most likely be lower. First trials on large representative patient populations or patients with an increased risk of colorectal cancer are therefore essential.

## 5. Identification of Biomarkers

The first studies investigating the possibility of early diagnosis of breast and colorectal cancer with mass spectrometry did not include identification of discriminating peaks. Ideally, these would all be proteins produced by tumor cells only and secreted into the blood in sufficient quantities to be detected. The identification of discriminatory proteins has become an important element in recent studies and will be discussed below.

Identifying proteins is by no means simple and requires additional analytical tools. In the early days, MALDI-TOF mass fingerprinting was used for MS-based protein identification. To this end, a protein is enzymatically converted into peptides, typically with trypsin. Since the tryptic digestion is highly site-specific, the identification of at least two peptides allows identification of the original protein. This method, however, only works for purified proteins. Nowadays, the method of choice for protein identification is tandem mass spectrometry (MS/MS or MS^2^). The tryptic peptides are first separated using high-performance liquid chromatography (HPLC) before performing MS/MS identification. The HPLC is interfaced with a tandem mass spectrometer through an electrospray ionization (ESI) source. So-called LC-MS/MS methods are highly suited and optimized for peptide sequencing. Sequencing experiments (*i.e.*, MS/MS) are carried out on ions that are selected in a prescan (*i.e.*, MS). Peptides are collided with inert gas which causes these peptides to fragment, resulting in product ions that can be interpreted with respect to the primary amino acid sequence. The resulting spectra are used to identify the peptides in question. This can be done in various ways; by *de novo* sequencing or by spectral matching using databases. With *de novo* sequencing, the amino acid sequence of a peptide is reflected in the fragment ion mass spectrum. The mass difference between two neighboring peaks is equal to the mass of one amino acid. However, when not all peptide bonds are broken, or when all expected fragment ions do not appear in the mass spectrum, interpretation may be ambiguous. Therefore, spectral matching is more frequently used. This method identifies peptides by comparing an MS/MS-spectrum with theoretically expected peptide spectra that are stored in a database. After comparison, the best matching peptide(s) are given together with a score indicating the closeness of the match. This database (SpectraST for example) consists of a collection of theoretical spectra that are derived from all possible proteins that can originate from the genome. These databases take certain splice-variants into account; however the existence of alternative splice variants or mutations, related for instance to cancer, hampers the identification of peptides. A related problem with this approach is the redundancy of proteins that do not actually occur, and thus the chance of accidental matches is increased. Another possibility, is matching the spectrum of product ions to spectra that were obtained from standard (synthetic) or previously identified peptides. This approach has the advantage that the database does not include any proteins that are not naturally present and thus decreases the number of false positives. Obviously, the disadvantage is that it cannot be used to identify proteins that are not included in the database. Note the difference between peptide and protein identifications, that is, the peptides are identified directly from the MS/MS-spectra, with a certain confidence, whereas a protein identification is derived from a combination of multiple peptide ID’s. Several parameters exist to express the reliability of the peptide and resulting protein matches. The mathematics and statistics that are used for this purpose fall beyond the scope of this review but are reviewed elsewhere [72]. The reliability of protein identification can be increased by using known properties of the yet unidentified protein. For instance, if the protein is also analyzed on SDS-PAGE, its mass can be identified and proteins that have a different mass can be left out of the database analysis. Some studies have used western blotting to identify proteins on SDS-PAGE after identification which is an effective method to confirm protein identity, if reliable antibodies are available.

### 5.1. Biomarker Identification in Breast Cancer

Only limited studies have identified biomarkers in MS studies for breast cancer. Li *et al*. tried to identify their previously identified BC1-3, but could only identify BC2 and BC3 as fragments of C3a, desArg [62]. This protein was also identified in colorectal and MS studies in other forms of cancer. The BC1 is suspected to be an interalpha-trypsin inhibitor heavy chain H4. Fan *et al*. found apolipoprotein C-I to be down-regulated in breast cancer patients. The two other discriminatory peaks were identified as C-terminal-truncated form of C3a and its complement component C3a [53]. As in colorectal studies, the previously identified proteins might seem to be lacking in specificity as these are not tumor-produced proteins. However, Villanueva *et al*. described not only cancer-specific, but cancer type-specific biomarkers [63]. The strength of this study was that it was the first, not only to take the identity of the potential biomarkers into account, but also to realize the importance of the biomarkers’ mass. This study found 11 unique biomarkers for breast cancer compared to prostate cancer and bladder cancer patients. These were all protein fragments cleaved from proteins normally present in the serum (fibrinogen α, C4a, C3f, ITIH4, ApoA-IV and transthyretin). Further research into these 11 biomarkers might find a set of unique biomarkers for breast cancer. It therefore seems that the biomarkers that are discovered with MS are not tumor-specific proteins, but tumor-specific protein fragments. This may well be due to tumor-specific secretion of proteases which cleave high-abundant serum proteins (Table 3).

### 5.2. Biomarker Identification in Colorectal Cancer

One of the most frequently found potential biomarkers, C3a-desArg, is not a tumor secreted protein, but a component of the complement system. Elevation of this protein is therefore more likely to be a reflection of the body’s inflammatory response activated by cancer. Interestingly, using serum ELISA testing of C3a-desArg levels, Habermann *et al*. were able to identify cancer patients with a sensitivity of 96.8% and specificity 96.2%. However the control group in this study consisted of healthy medical personnel. This group was not age matched and might therefore have a relatively lower chance of additional diseases than a screening population aged 50–75 years, which might lead to nonspecific elevation of C3a-desArg levels. For instance, Li *et al*. also reported an elevation of C3a-desArg in patients with breast cancer [62]. This implies that the elevation of these proteins is in fact non-specific and has little value in early identification of colorectal (or breast cancer) [73]. Another identified protein, by Ward *et al*., was a peak at m/z ratio 5070 Da, which was identified as α1-antitrypsin and is involved in the immune response. It has also been implicated in other forms of cancer, so this is unlikely to be a specific indicator of CRC. Albrethsen *et al*. found an increase in serum human neutrophil peptides 1, 2 and 3 (HNP 1–3) signals compared to controls via Seldi-TOF mass spectrometry. These proteins are involved in regulation of the immune response. HNP 1–3 are found to be upregulated in colorectal cells compared to normal epithelial cells [74]. Testing for CRC by measuring serum levels with an ELISA assay, yielded a sensitivity of 69% and specificity of 100%, in a group of 26 colon cancer patients and 22 controls. However, the control group consisted of healthy controls only. Because of this, the high specificity is likely to be overoptimistic. Expression of HNP 1–3 has been found in a variety of other tissues, both in inflammatory and neoplastic conditions. Engwegen *et al*. found a non-specific increase of discriminating proteins (N-terminal fragment of albumin, apolipoprotein CI, apolipoprotein AI and a yet unidentified protein at 5900 kDa) in other cancer types as well. However, some of these acute phase proteins might be used in combination with other biomarkers that are more specific biomarkers for CRC. For instance, the m/z ratio 5900 Da peak also found by Engwegen *et al*. (and by Yu *et al*.) was able to discriminate 76% of CRC from other forms of cancer. So far this protein has not been identified (Table 4).

## 6. Discussion

Numerous studies have described favorable reports on serum protein profiling of breast and colorectal cancer patients. These studies used limited amounts of patients and were generally case-control studies. The fact that the control groups consisted of healthy people has made it impossible to determine whether discriminatory peaks are actually cancer-specific or only “disease-specific”. It may be that peaks that are now seen as cancer-specific are in fact due to inflammation or obstruction caused by cancer. Further studies, not only including healthy persons, but also a control group representative of the general patient population, are essential to help to resolve this question. Unfortunately most reports lack detailed information regarding the control group used.

In addition, prospective studies are needed to determine the value of MS in clinical practice. However, before these can take place, more research needs to be done on the reproducibility and optimal handling and processing methods [76]. In our opinion, an ideal set up to apply MS in a routine clinical screening setting would be to first validate the profiles in a population screening. Secondly, centralized profiling could be performed in, for example, specialized regional centers. Finally, when discriminating proteins are identified, a simple test (e.g., ELISA) could replace profiling for the identification of cancer patients.

Studies on serum samples have identified several potential biomarkers. Most of the markers that have been identified so far were (cleaved) proteins that were present in the serum at relatively high concentrations, *i.e.*, the so-called high abundant proteins (milli-microgram/mL) [77]. In this respect, MS faces the challenge of the high dynamic concentration range since tumor-specific proteins are often low abundant (<100 nanogram/mL). In addition, there are indications that the entire spectrum of cleaved proteins by tumor-specific exoproteases can be used to identify patients with cancer. This implies that not only the identity, but also the size of the biomarker, is important for accurate diagnosis [78]. Only testing for the presence of a certain biomarker is likely to be nonspecific, since this protein might also present in other diseases and other forms of cancer. However, the spectrum of specific fragments of these proteins might be the key to a successful diagnosis instead of conventional single biomarkers. Ironically, the breakdown of these proteins occurs after collection of the serum sample from the patient. This makes it all the more important to have strict guidelines for handling the samples after collection, if results are to be reproducible between different centers. All of these results have changed the way of thinking about biomarkers. Finding a single biomarker with MS might be impossible, since all tumors have a different molecular background, but it might be possible to combine several protein fragments to develop highly reliable tests allowing early cancer diagnosis. Although there are doubts about some of these results [79], MS remains a powerful tool in propelling these discoveries into the clinical practice.

## 7. Conclusion

In conclusion, several methods exist for the early diagnosis of colorectal and breast cancer. Current screening methods have disadvantages such as high-cost, be of an invasive nature, or offer insufficient sensitivity or specificity. Because of this, the search for a better diagnostic screening test for both these types of cancer is still ongoing. MS has recently been applied for identifying serum biomarkers and may lead to a relatively inexpensive (approximately 15 € per sample), minimally-invasive and reliable test, for early cancer diagnosis.

Several case-control studies have reported favorable results for diagnosis of breast and colorectal cancer. Comparing the reported sensitivities and specificities of the different research groups with current screening techniques, MS would appear to be very promising, however, screening results based on these groups, due to the increased *a priori* chance, are likely to be overoptimistic when compared to screening in a normal population. In addition, these studies used different methods, handling protocols, and significantly altered peaks for discriminating between cancer patients and healthy controls. In order to apply MS in a routine clinical setting, collecting, measuring and processing of data will need to be subject to stringent quality control procedures. The current roboting techniques allow high throughput. More comparative studies on influential factors and optimal methods are necessary. Subsequent prospective studies in representative patient populations can then determine whether MS is superior to other screening methods.

## Figures and Tables

**Table 1.** Early detection of breast cancer.

**Study**	**MS Method**	**Study Size N =**	**Sensitivity**	**Specificity**	**External Validation?**
Hu *et al*. The Breast 2005 [52]	SELDI-TOF	49 BC 51 BBD 33 HC	83.33%	88.89%	Yes N = 18 BC, 9HC
Fan *et al*. Journal of Cancer Research and Clinical Oncology 2010 [53]	SELDI-TOF	80 BC 40 HC	96.45%	94.87%	Yes N = 44 BC, 98 BBD, 20 HC
Belluco *et al*. Annals of Surgical Oncology 2007 [54]	SELDI-TOF	109 BC 109 HC	95.6%	86.5%	Yes N = 46 BC, 46 HC
Callesen *et al*. Journal of Proteome Research 2008 [55]	SELDI-TOF	48 BC 28 HC	85%	85%	No
Li *et al*. Clinical Chemistry 2002 [56]	SELDI-TOF	103 BC 25 BBD 41 HC	93%	91%	No
Vlahou *et al*. Clinical Breast Cancer 2003 [57]	SELDI-TOF	45 BC 42 BBD 47 HC	80%	79%	No
De Noo *et al*. Onkologie 2006 [45]	MALDI-TOF	78 BC 29 HC	100%	97%	No

BC = Breast cancer, BBD = Benign breast disease, HC = Healthy controls.

**Table 2.** Early detection of CRC.

**Study**	**MS Method**	**Study Size (N)**	**Sensitivity**	**Specificity**	**External validation?**
Yu *et al*. World J Gastroenterology 2004 [64]	SELDI-TOF	55 CRC 35 CRA 92 HC	89%	83–92%	No
Liu *et al*. Cancer Investigation 2006 [65]	SELDI-TOF	74 CRC 48 HC	95%	94.87%	Yes N = 60 CRC, 39 HC
De Noo *et al*. European Journal of Cancer 2006 [45]	MALDI-TOF	66 CRC 50 HC	95.2%	90.0%	No
Ward *et al*. British Journal of Cancer 2006 [67]	SELDI-TOF	62 CRC 31 HC	95%	91%	No
Chen *et al*. Clinical Cancer Research 2004 [69]	SELDI-TOF	55 CRC 92 HC	91%	93%	No
Zhao *et al*. Chinese Journal of Clinical Medicine 2004 [70]	SELDI-TOF	73 CRC 16 CRA 31 HC	96%	98%	Yes N = 73 CRC, 16 CRA, 31 HC
Engwegen *et al*. World Journal of Gastroenterology 2006 [68]	SELDI-TOF	77 CRC 80 HC	66.7–89.5%	73.3–88.9%	Yes

CRC = Colorectal adenoma, CRA = Colorectal adenoma, HC = Healthy controls.

**Table 3.** Biomarker identification in breast cancer.

**Author**	**Identified biomarkers (m/z ratio)**
Li *et al*. Clinical Chemistry 2005 [62]	C3 fragment (8.1 × 10^3^/8.9 × 10^3^)
Fan *et al*. Journal of Cancer Research and Clinical Oncology 2010 [53]	Apolipoprotein C-I (6.6 × 10^3^) C3 fragment (8.1 × 10^3^/8.9 × 10^3^)
Villanueva *et al*. Journal of Clinical Investigation 2006 [63]	FPA, fibrinogen alpha, C3f, C4a, ITIH4, ApoA-IV, Bradykinin, Factor XIII, Transthyretin

**Table 4.** Biomarker identification in colorectal cancer.

**Author**	**Identified biomarkers (m/z ratio)**
Engwegen *et al*. World Journal of Gastroenterology 2006 [68]	-N-terminal albumin fragment (3.1 × 10^3^) -Apolipoprotein C-I (3.3 × 10^3^/6.6 × 10^3^) -Apolipoprotein A-I (28 × 10^3^)
Ward *et al.* British Journal of Cancer 2006 [67]	-Alpha1-antitrypsin (50.7 × 10^3^) -Apolipoprotein C-I (6.4 × 10^3^/6.6 × 10^3^) -Transferrin (79.1 × 10^3^), -C3 fragment (8.94 × 10^3^)
Albrethsen *et al*. BMC Cancer 2005 [75]	-HNP 1 (3.37 × 10^3^) -HNP 2 (3.44 × 10^3)^) -HNP 3 (3.49 × 10^3^)
